# INSC Is Down-Regulated in Colon Cancer and Correlated to Immune Infiltration

**DOI:** 10.3389/fgene.2022.821826

**Published:** 2022-05-19

**Authors:** Tao Yu, Dan Li, Zhi Zeng, Xu Xu, Haiming Zhang, Jie Wu, Wei Song, Hua Zhu

**Affiliations:** ^1^ Department of Oncology, Integrated Chinese and Western Medicine, The Central Hospital of Wuhan, Tongji Medical College, Huazhong University of Science and Technology, Wuhan, China; ^2^ Department of Pharmacy, Renmin Hospital of Wuhan University, Wuhan, China; ^3^ Department of Pathology, Renmin Hospital of Wuhan University, Wuhan, China; ^4^ Department of Geriatrics, Renmin Hospital of Wuhan University, Wuhan, China; ^5^ Department of Neurosurgery, Renmin Hospital of Wuhan University, Wuhan, China

**Keywords:** INSC, immune cell infiltrates, prognosis, colon cancer, bioinformatics analysis

## Abstract

**Background:** Previous studies have verified that Inscuteable Spindle Orientation Adaptor Protein (INSC) can regulate cell proliferation and differentiation in the developing nervous system. It also plays an important role in spindle orientation during mitosis and asymmetric division of fibroblasts and participates in the process of stratification of the squamous epithelium. The role and potential mechanism of INSC in the development of colonic adenocarcinoma (COAD) have not been fully understood. This study aimed at exploring the prognostic value of INSC in COAD and the correlation of its expression with immune infiltration.

**Methods:** The Cancer Genome Atlas (TCGA), Genotype-Tissue Expression (GTEx) project, Gene Expression Profiling Interactive Analysis (GEPIA), and Gene Expression Omnibus (GEO) database were used to analyze the expression of INSC in COAD. The INSC protein expression level was analyzed by immunohistochemistry staining and the Human Protein Atlas (HPA) database. The diagnostic and prognostic values of INSC in COAD patients were analyzed using receiver operating characteristic (ROC) and Kaplan–Meier (KM) survival curves. In order to understand whether INSC is an independent prognostic factor, we used univariable and multivariate Cox analyses to analyze INSC expression and several clinical characteristics with survival. We use STRING analysis to find INSC-related proteins and related biological events analyzed by Gene Ontology (GO) annotation and Kyoto Encyclopedia of Genes and Genomes (KEGG) analysis. At last, GEPIA and the Tumor Immune Estimation Resource (TIMER) were employed to explore the relationship between INSC and immune infiltrates and its marker gene set.

**Results:** INSC was lower expressed in COAD tissues than in normal colon tissues, which was correlated with tumor stage. Patients with lower expression of INSC had shorter overall survival (OS). Moreover, univariable Cox analysis demonstrated that high expression of INSC was an independent prognostic factor for COAD. ROC analysis showed INSC was an accurate marker for identifying tumors from normal colon tissue, and the AUC of the curve was 0.923. Significant GO term analysis by GSEA showed that genes correlated with INSC were found to be enriched in several immune-related pathways. Specifically, INSC expression showed significant negative correlations with infiltration levels of B cells, CD4^+^ T cells, macrophages, DCs, and their marker sets in COAD.

**Conclusion:** INSC was provided with prognostic value in COAD and related to immune invasion.

## 1 Introduction

Colonic adenocarcinoma (COAD) is one of the most prevalent digestive tract cancers, with a significant fatality rate (Sanoff et al., 2007). Worryingly, the rates of recurrence and death of COAD are rising (Bray et al., 2018). Despite recent advancements in therapy, the 5-year survival rate has not increased appreciably. As a result, finding gene signatures or biomarkers to detect the intrinsic genetic and epigenetic heterogeneity of COAD, as well as developing prognostic models to guide therapy, is critical.

In terms of morbidity, colon cancer, a malignancy of the alimentary canal, ranks third among malignant tumors globally (Bray et al., 2018). According to recent research, more than one million people are diagnosed with colon cancer each year, with a disease-specific death rate of over 33% in industrialized nations (Ferlay et al., 2015). Colon cancer mortality is on the rise as a result of dietary and lifestyle changes (Mcguire, 2016). Despite significant improvements in colon cancer treatment choices, the 5-year survival rate remains poor. As a result, we must seek novel biomarkers to aid in the correct and early diagnosis of COAD, as well as identify effective targets to increase the treatment impact.

INSC (INSC Spindle Orientation Adaptor Protein) is a protein-coding gene according to previous studies. Apico-basal polarity in epithelial stem cells is produced by apical enrichment of the polarity proteins Par3: Par6: aPKC, which can recruit an adaptor called Inscuteable at the apical membrane. INSC codes for a conserved 35-residue peptide (INSC PEPT hereon) that binds to the N-terminal TPR domain of LGN/dLGN with nanomolar affinity 10, 11, and 19 (Culurgioni et al., 2018).

The INSC-dependent system, which includes INSC, BAZ, and PINS, is active throughout mitosis, whereas the cryptic INSC-independent pathway is functional only late in mitosis (anaphase and telophase) and is essential for telophase rescue (Wang et al., 2006). The mitotic spindle is also directed to align along the polarity axis by INSC (Bowman et al., 2008). Current findings in the mouse *epidermis* reveal that the protein INSC plays a critical function in appropriate spindle orientation as both an instructional and regulatory signal (Poulson and Lechler, 2010), which keeps the polarity of the Par complex and the neuroblast (Schober et al., 1999). A genome-wide association study identified INSC gene was associated with Alzheimer’s disease-related cognitive phenotypes (Wang et al., 2021). However, the role of the INSC gene in COAD malignancy has not been reported.

In the present study, public databases such as The Cancer Genome Atlas (TCGA) and Gene Expression Omnibus (GEO) were used to comprehensively investigate the relationship between INSC and the prognosis of COAD. TIMER was employed to assess the correlation between INSC and tumor immune cell infiltration. The results provided new insights into the function of INSC and novel targets for the COAD diagnosis and prognosis.

## 2 Methods

### 2.1 RNA-Sequencing Data and Bioinformatic Analysis

Expression data of INSC gene and clinical information of COAD patients were collected from The Cancer Genome Atlas (TCGA, https://tcga.xenahubs.net), Genotype-Tissue Expression (GTEx) project (https://gtexportal.org/) (GTEx Consortium, 2020), and Gene Expression Omnibus (GEO) (http://www.ncbi.nlm.nih.gov/geo/) database (Barrett et al., 2013). The data of 521 samples, including 41 para-cancerous tissues and 480 tumor tissues, from TCGA were extracted. There were 308 normal colonic tissues obtained from GTEx. Additionally, the INSC expression data were obtained from GSE44076 and GSE39582 datasets in the GEO database to verify the INSC expression level in tumor and non-tumor tissues. The boxplot was realized by using the R software package “ggplot2.”

### 2.2 Protein Expression Analysis

The human protein atlas (HPA) database (https://www.proteinatlas.org/) (Uhlén et al., 2015) was applied to determine INSC protein expression levels through immunohistochemistry (IHC) staining (antibody HPA039769). We obtained the INSC IHC photographs of COAD patients from the HPA database.

In order to further confirm the expression of INSC in the colon and COAD tissues, 82 paired normal and COAD tissues (49 men and 33 women, average age of 63 ± 13 years old) were enrolled and determined by IHC staining (Li et al., 2021a). Briefly, deparaffinized and rehydrated sections were subjected to 3% H_2_O_2_ and then antigen retrieval by citric acid buffer (pH 6.0). After sealing at room temperature for 20 min with 5% bovine serum albumin, the slices are incubated overnight (16–18 h) at 4°C with primary anti-INSC antibody (1:50 dilution for colon tissues, HPA039769, Atlas Antibodies AB, Sweden). Then, the sections were incubated with biotinylated-linked antibodies and peroxidase-labeled streptavidin (UltraSensitive™ SP (Mouse/Rabbit) IHC Kit-9710; Maixin Bio, Fujian, China) for 15 min at room temperature. Then, the reaction products were stained with 3,3′-diaminobenzidine (DAB) and lightly counterstained with hematoxylin. The sections with PBS instead of primary antibody served as a negative control. The INSC expression levels were evaluated according to the average score of two independent pathologists’ evaluations who were unaware of the diagnosis outcome. INSC expression in tumor cells was classified based on a four-tier grading system (scores: 0 = absent, 1 = weak, 2 = moderate, and 3 = strong staining). Generally, a score less than 1 was considered negative, and a score more than 1 was considered positive.

### 2.3 Prognosis Analysis of Inscuteable Spindle Orientation Adaptor Protein Expression in Colonic Adenocarcinoma

First, we applied the KM survival and receiver operating characteristic (ROC) curves to assess the prognostic values of INSC in patients with COAD. Second, we performed univariate and multivariate regression analyses to evaluate the relationship between the expression of INSC and the overall survival (OS) of patients with COAD as we previously reported (Zhu et al., 2021a; Li et al., 2021b). The forest realized through the “forestplot” R package was applied to display the *p*-value, hazard ratio (HR), and 95% confidence interval (CI) of each variable.

### 2.4 Functional Analysis of Inscuteable Spindle Orientation Adaptor Protein in Colonic Adenocarcinoma

The (protein–protein interaction) PPI network of INSC was conducted by the Search Tool for the Retrieval of Interacting Genes/Proteins (STRING) (version: 11.5, https://cn.string-db.org/) database. The minimum required interaction score was set as medium confidence 0.400. The max number of interactors to show is as follows: first shell: no more than 10 interactors; second shell: none. We use the R packs such as clusterProfiler [version 3.14.3] and Org Hs. Eg.db [version 3.10.0] to perform the Gene Ontology (GO) and the Kyoto Encyclopedia of Genes and Genomes (KEGG) pathway enrichment analysis of the obtained genes, and the *p*-value is set to be less than 0.05. The results are visualized and displayed in a bubble chart with the ggplot2 package [version 3.3.3].

We further used LinkOmics (http://www.linkedomics.org/login.php) (Vasaikar et al., 2018) to conduct the GO and KEGG pathway enrichment analysis. The cancer type: TCGA_COADREAD; data type: RNAseq; platform: HiSeq RNA; and target dataset: (data type: RNAseq; platform: HiSeq RNA).

### 2.5 Association Between Immune Cell Infiltration and Inscuteable Spindle Orientation Adaptor Protein in Colonic Adenocarcinoma

We used Tumor Immune Estimation Resource (TIMER) (https://cistrome.shinyapps.io/timer/) and Gene Expression Profiling Interactive Analysis (GEPIA) (http://gepia.cancer-pku.cn/api.html) to investigate the correlations between INSC and tumor-infiltrating immune cells (TIIC). Spearman correlation analysis and the CIBERSORT method were further employed to evaluate the correlation between INSC and TIIC and their corresponding molecular markers.

### 2.6 Statistical Analysis

The data analysis and mapping involved in this study are completed by R software (version 3.6.3) and R language package ggplot2 (version 3.3.3). RNAseq data were converted into TPM (transcripts per million reads) format, and log2 conversion is performed. For the comparison between groups, the normality test should be conducted first. If the samples do not meet the normality test (*p* < 0.05), then the Mann Whitney *U* test (Wilcoxon rank sum test) will be selected. The chi-square test was used to analyze the correlation of INSC expression with the clinicopathological factors of COAD. The Kaplan–Meier method was used to calculate the relationship between INSC expression and prognosis of COAD. Univariate and multivariate Cox regression analyses were conducted to assess the effect of INSC on the prognosis of COAD. The “survival” package (3.2-10 version) was applied for statistical analysis of survival data, and the “survminer” package (0.4.9 version) was employed for mapping survival curves. The forest was applied to show the *p*-value, HR, and 95% CI of each variable. Spearman correlation analysis was used for genetic correlation. *p* ≤ 0.05 was considered to be statistically significant.

## 3 Results

### 3.1 Association Between the Expression of Inscuteable Spindle Orientation Adaptor Protein and Clinicopathological Features in Colonic Adenocarcinoma Patients

The clinicopathological features of 478 COAD patients were extracted from TCGA, including age, gender, TNM stage, pathologic stage, and CEA level. According to the expression level of INSC, we divided all samples into high expression and low expression INSC groups by median. Detailed clinical information was shown in Supplementary Table S1.

### 3.2 Expression of Inscuteable Spindle Orientation Adaptor Protein mRNA in Colonic Adenocarcinoma

**FIGURE 1:**
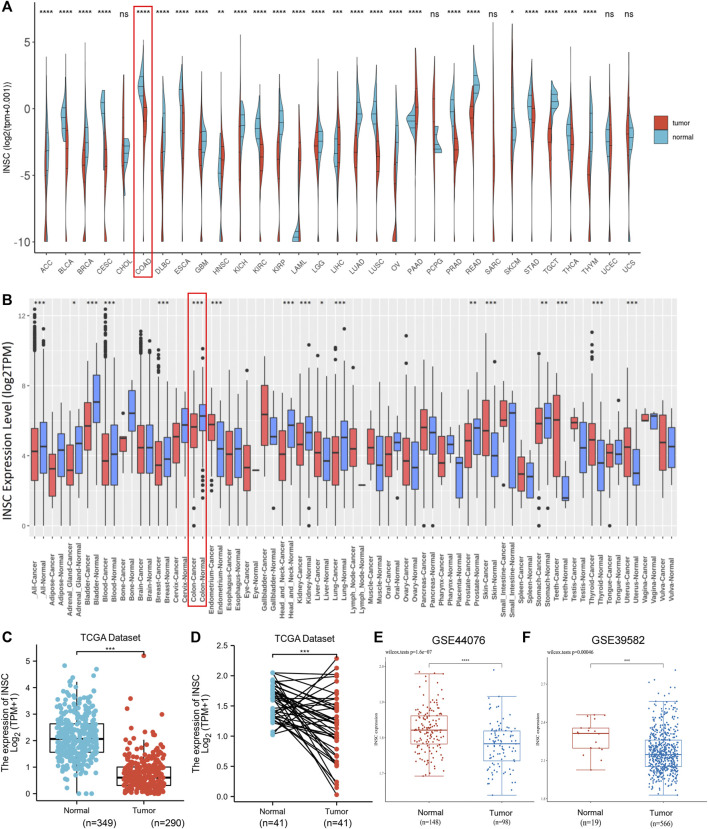
INSC expression levels in cancers. **(A–B)** The expression levels of INSC were different in pan-cancer tissues compared to those in normal tissues according to TCGA datasets **(A)** and GEO datasets **(B)**, respectively. **(C–D)** Compared to the normal colon tissue, the expression level of INSC was significantly decreased in COAD tissues (*p* <0.001). **(C)** Unpaired samples in TCGA datasets and GTEx datasets. **(D)** Paired samples in only TCGA datasets. The expression of INSC from GSE44076 **(E)** and GSE39582 **(F)** in GEO datasets (*p < 0.05, **p <0.01, ***p < 0.001, ****p < 0.0001).

We investigated numerous sets of data from different databases in order to better understand INSC expression in tumors and normal tissues. The GEPIA database was used to validate INSC mRNA expression. The data are presented as a body heat map (Supplementary Figure S1). The color red denotes cancerous tissue, whereas the color green denotes normal tissue. The higher the intensity of expression, the darker the hue. The region around the large intestine is brightly colored, showing that INSC is abundantly expressed in a healthy large intestine. The differential expression of INSC mRNA in pan-cancer cells was next investigated. Figures 1A and B show the data from TCGA and GEO, respectively. It was found that in adrenocortical carcinoma, breast cancer, bladder cancer, colon cancer, head and neck cancer, kidney cancer, stomach adenocarcinoma, prostate cancer, lung cancer, and COAD, the INSC mRNA expression was lower in tumors than in normal tissues. Figures 1C–F illustrate the mRNA expression of INSC in COAD from TCGA and GEO. INSC expression was considerably lower in COAD tissues than in normal tissues (*p* < 0.001).

### 3.3 Protein Expression of Inscuteable Spindle Orientation Adaptor Protein in Colon Normal and Cancer Tissues

**FIGURE 2:**
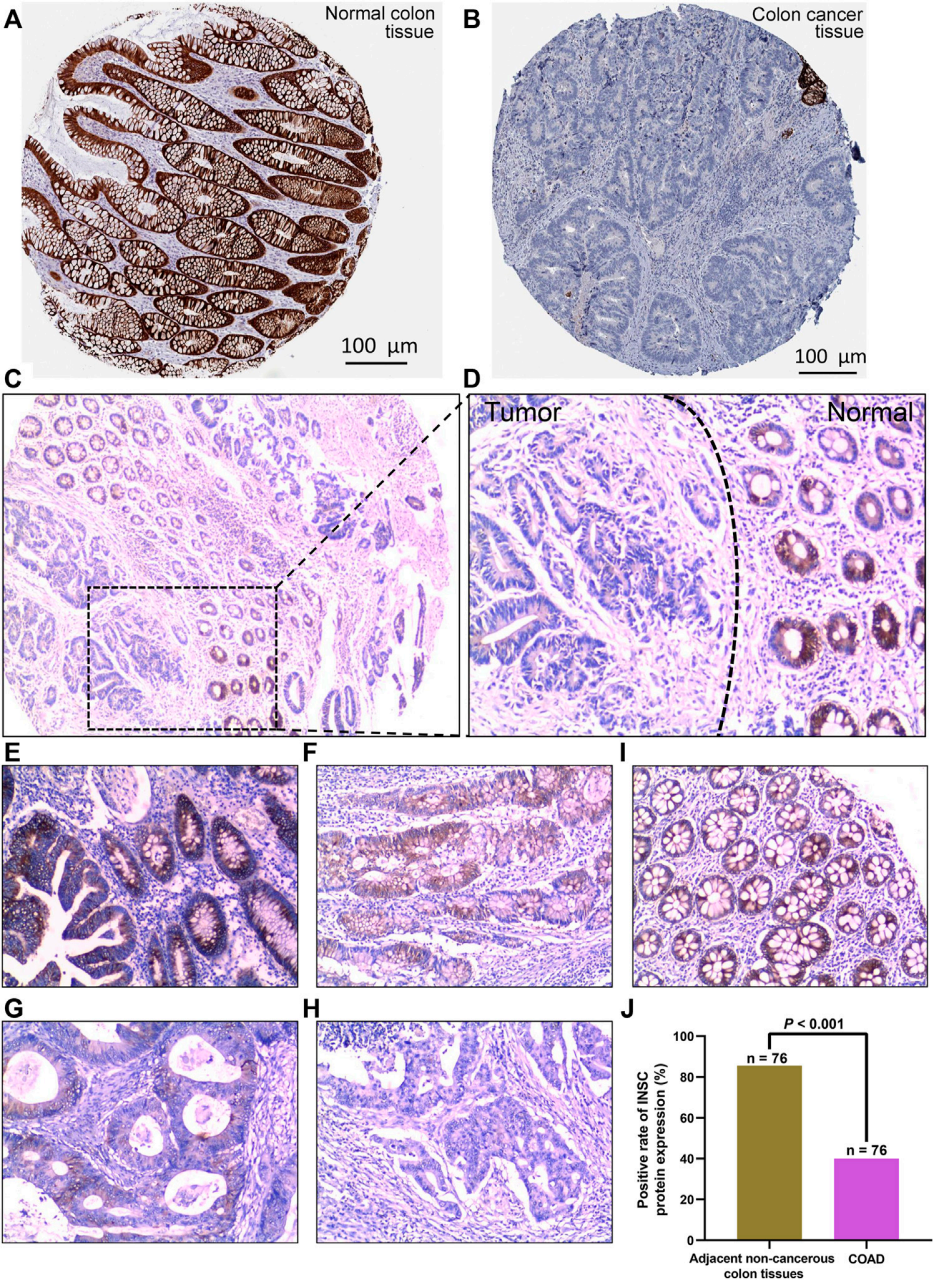
INSC expression in normal colon and COAD tissues was determined by immunohistochemistry. The level of INSC protein in COAD **(A)** and normal tissues **(B)** (Antibody HPA039769) form the HPA database; **(C)** INSC protein expression in COAD and non-cancerous colon tissues and its immunohistochemical staining in representative tissue specimens. Original magnification, ×40; **(D)** immunohistochemical staining of INSC proteins in representative COAD and non-cancerous colon specimens. The left side represents cancer tissues and the right side represents non-cancer tissues. Original magnification, ×100; **(E–H)** the score of INSC expression in representative tumor cells of COAD ((E) score = 3, **(F)** score = 2, **(G)** score = 1, and **(H)** score = 0); magnification, ×100; **(I)** expression of INSC in representative normal colon cells ((I) score = 3); magnification, ×100; **(J)** positive rate of INSC protein expression in adjacent non-cancerous colon tissues and COAD tissues (*p* <0.001, cancer *vs*. non-cancerous tissues, chi-squared test), *n* = 82, and six samples were lost.

The protein expression of INSC was analyzed by immunohistochemical staining images obtained from HPA. INSC protein was highly expressed in colonic mucosal glandular epithelial cells located in cytoplasmic and membranous (Figure 2A). On the contrary, there was less expression of INSC protein in COAD tissue (Figure 2B). To further investigate the protein expression of INSC in non-cancerous colon and COAD tissues, the INSC protein expression was detected in a larger size of 76 COAD tissues and its adjacent non-cancerous colon tissues by IHC staining. The results showed that non-cancerous colon tissues had stronger IHC staining than COAD tissues (Figures 2C, D). The scores of INSC protein expression ranging from 3, 2, 1, and 0 in representative COAD tissue specimens were presented in Figures 2E–H. The representative IHC staining of non-cancerous colon tissue with a score of 3 was shown in Figure 2I. It was also found that the positive expression rate of INSC was higher in normal colon tissues (85.53%) than that in COAD tissues (40.00%) (*p* < 0.001, Figure 2J).

### 3.4 Prognostic Potential of Inscuteable Spindle Orientation Adaptor Protein in Colonic Adenocarcinoma

**FIGURE 3:**
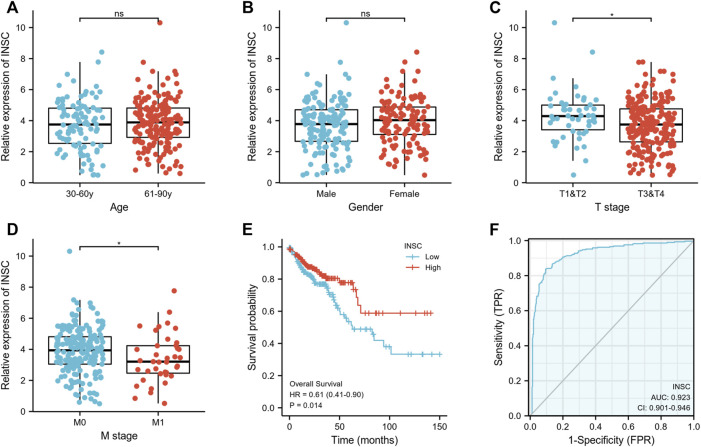
**(A,B)** Association between INSC expression and clinical features such as age and gender in COAD. **(C,D)** The association between INSC expression and clinical features such as T and M stages in COAD. Lower INSC expression was related to higher T and M stages (*p < 0.05). **(E)** The KM survival curve displayed a low level of INSC with a poor prognosis for COAD patients. **(F)** ROC analysis illustrated that INSC was an accurate marker to distinguish tumor from normal tissue. The AUC was 0.923.

**FIGURE 4:**
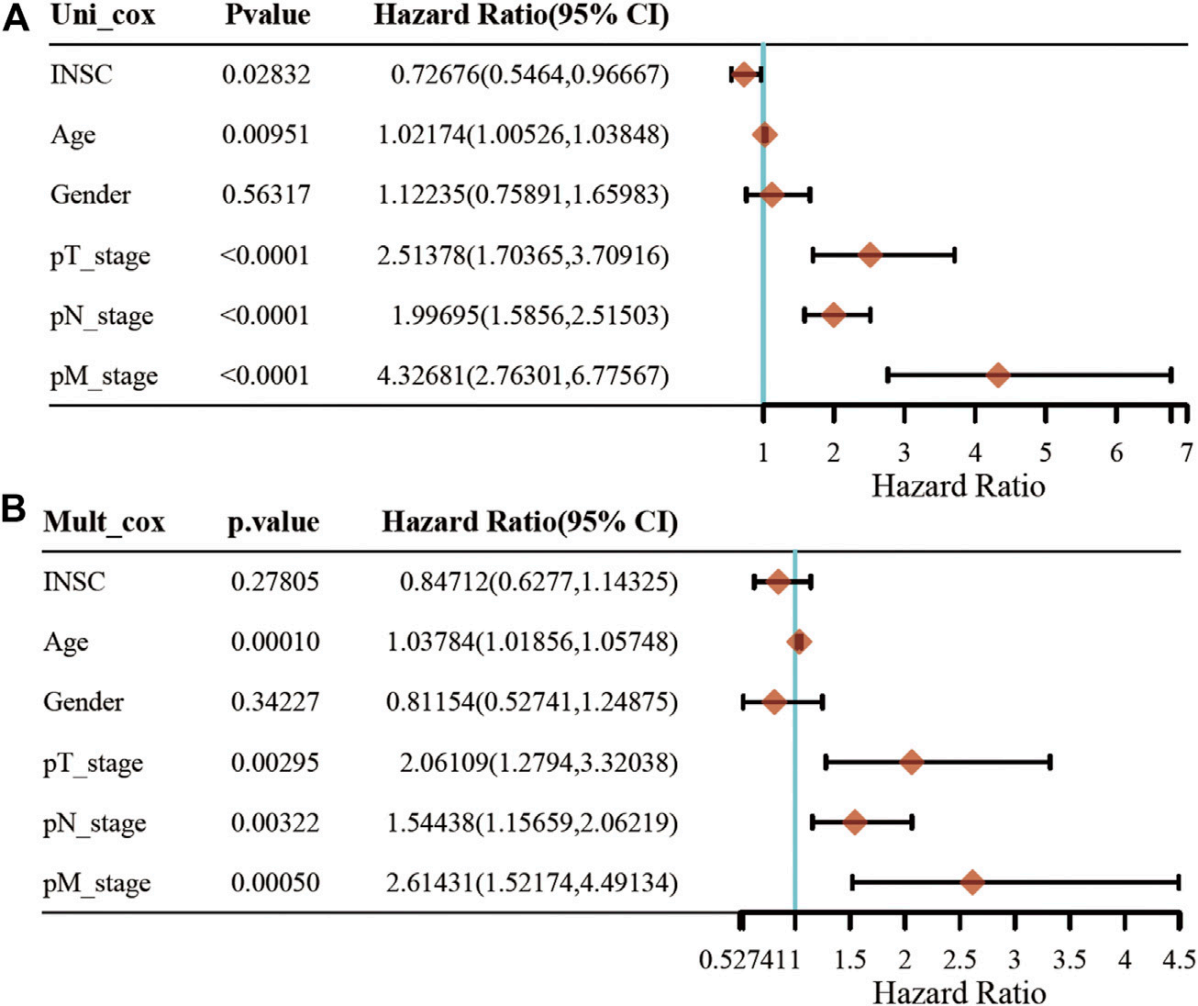
Univariate and multivariate Cox regression of the INSC gene and other five clinical characteristics. **(A)** The forest plot of univariate regression analysis. **(B)** The forest plot of multivariate regression analysis.

First, we explored the association of INSC with clinical manifestation in COAD. Lower INSC expression was shown to be related to greater T and M stages (*p* <0.05) but not age and gender (Figures 3A–D). The connection between INSC expression and survival outcomes was assessed by the KM survival curve. The median was used as the cutoff value for the high and low INSC expression groups. OS was shown to be longer in patients with increased INSC expression (log-rank test, *p* = 0.014) (Figure 3E). The ROC curve results were reported as AUC scores (area under the ROC curve) (Figure 3F). The AUC in this trial was 0.923 (CI: 0.901–0.946). INSC was able to discriminate between normal and malignant tissues according to these findings. Figures 4A, B showed a univariable and multivariable Cox regression analysis of INSC and clinical data that might be linked to OS. In the univariate Cox model, a low INSC level (*p* = 0.028) was linked with age and pathologic TNM stage in OS occurrences (*p* < 0.01). Age and pathologic TNM stage were independent variables associated with OS in COAD (*p* <0.01) in the multivariate Cox model.

### 3.5 Protein–Protein Interaction Networks and Enrichment Analyses of Inscuteable Spindle Orientation Adaptor Protein-Related Genes

**FIGURE 5:**
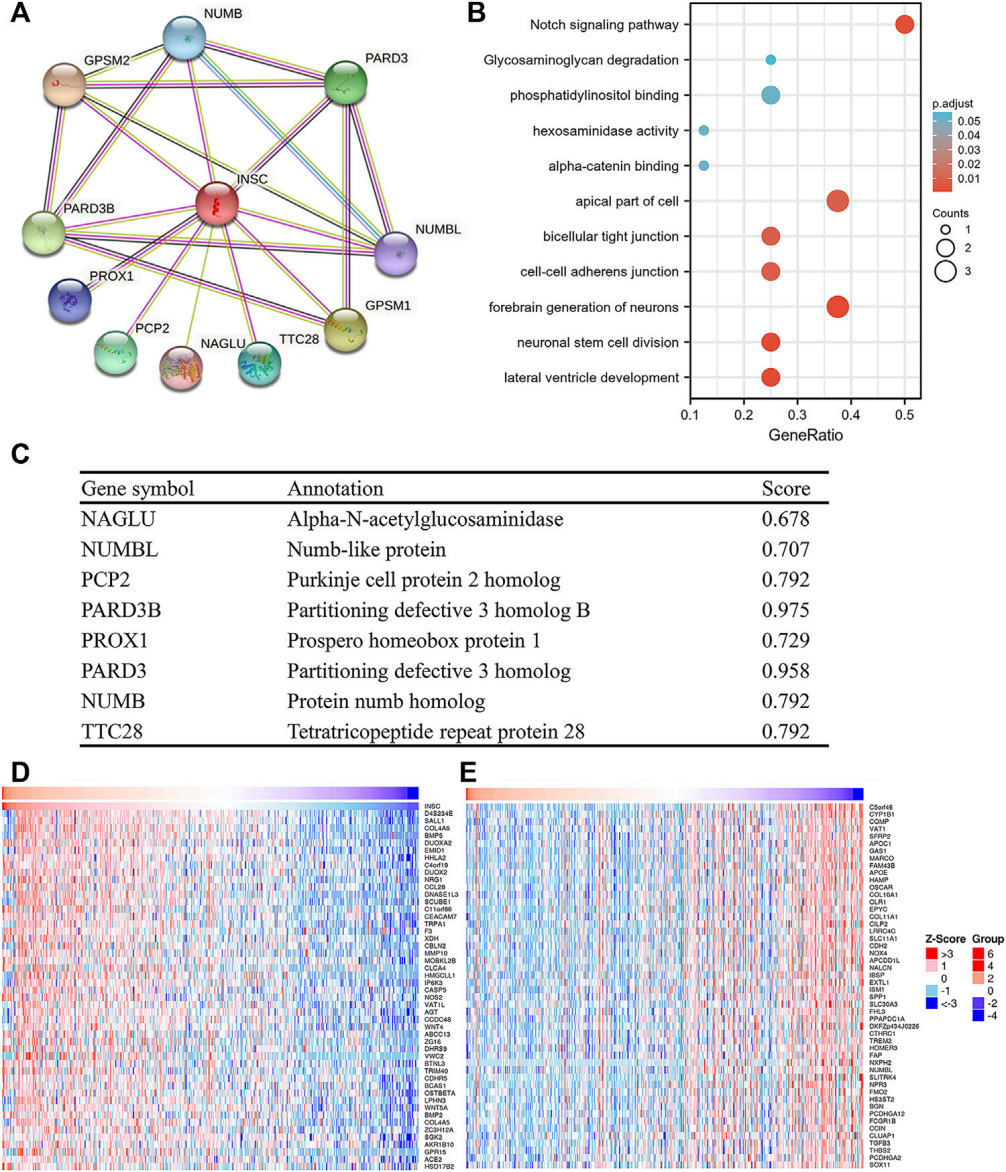
PPI and GO/KEGG analysis of INSC. **(A)** PPI network of INSC. **(B)** Bubble chart showing the enrichment results. **(C)** The detailed information of INSC-related genes. **(D)** Top 50 positively or negatively significant correlated genes with INSC.

**FIGURE 6:**
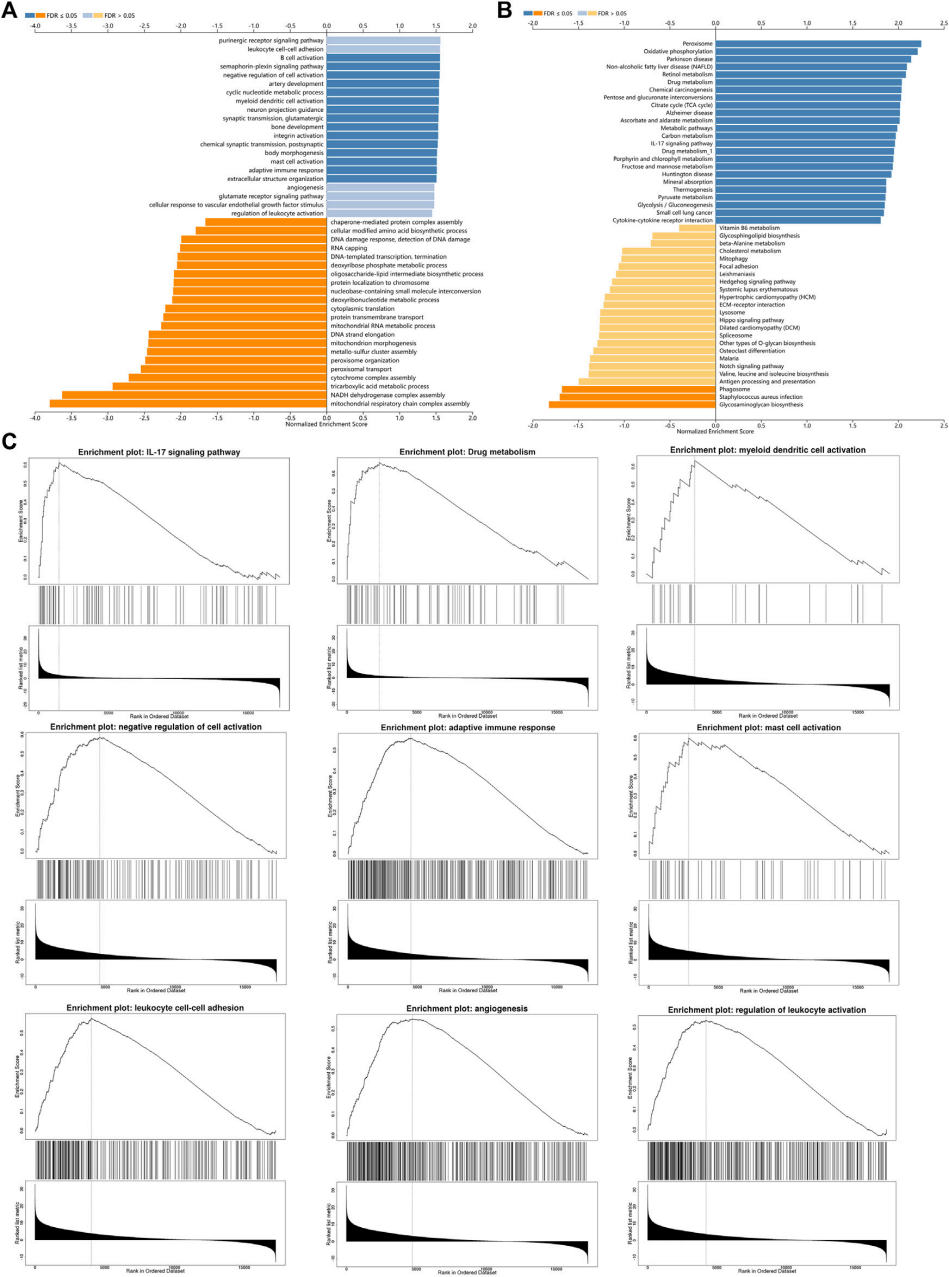
GO **(A)** and KEGG **(B)** pathway analyses from LinkOmics. **(C)** Immune-related biological processes and pathways that related to INSC in COAD.

In order to study the functional network of INSC-related genes in COAD, we first identified INSC-related genes with the STRING webtool. INSC-related genes included NAGLU, NUMBL, PCP2, PARD3B, PROX1, PARD3, NUMB, and TTC28 (Figure 5A). Their annotation and combined scores are presented in Figure 5C. Combined with the GO/KEGG enrichment analysis, we found that genes correlated with INSC were located mainly in the apical part of the cell (*p* < 0.001), cell–cell adherens junction (*p* < 0.001), and bicellular tight junction (*p* = 0.023). They were involved in the biological process of forebrain generation of neurons, lateral ventricle development and neuronal stem cell division. KEGG pathway analysis showed they were enriched in the Notch signaling pathway and glycosaminoglycan degradation (Figure 5B). The top 50 positively and negatively correlated genes with INSC were presented in Figures 5D, E. We further assessed the GO biological process and KEGG pathway analysis from LinkedOmics. The results showed that INSC was related to several immunological processes and pathways. The GO biological process analysis revealed that INSC was related to leucocyte cell–cell adhesion, B cell activation, negative regulation of cell activation, myeloid dendritic metabolic process, mast cell activation, regulation of leukocyte activation, and adaptive immune response (Figures 6A,C). The KEGG pathway analysis showed that INSC was associated with the IL-17 pathway, drug metabolism, and Notch signaling pathway (Figures 6B, C).

### 3.6 Association Between Inscuteable Spindle Orientation Adaptor Protein Expression and Immune Cell Infiltration in Colonic Adenocarcinoma

**FIGURE 7:**
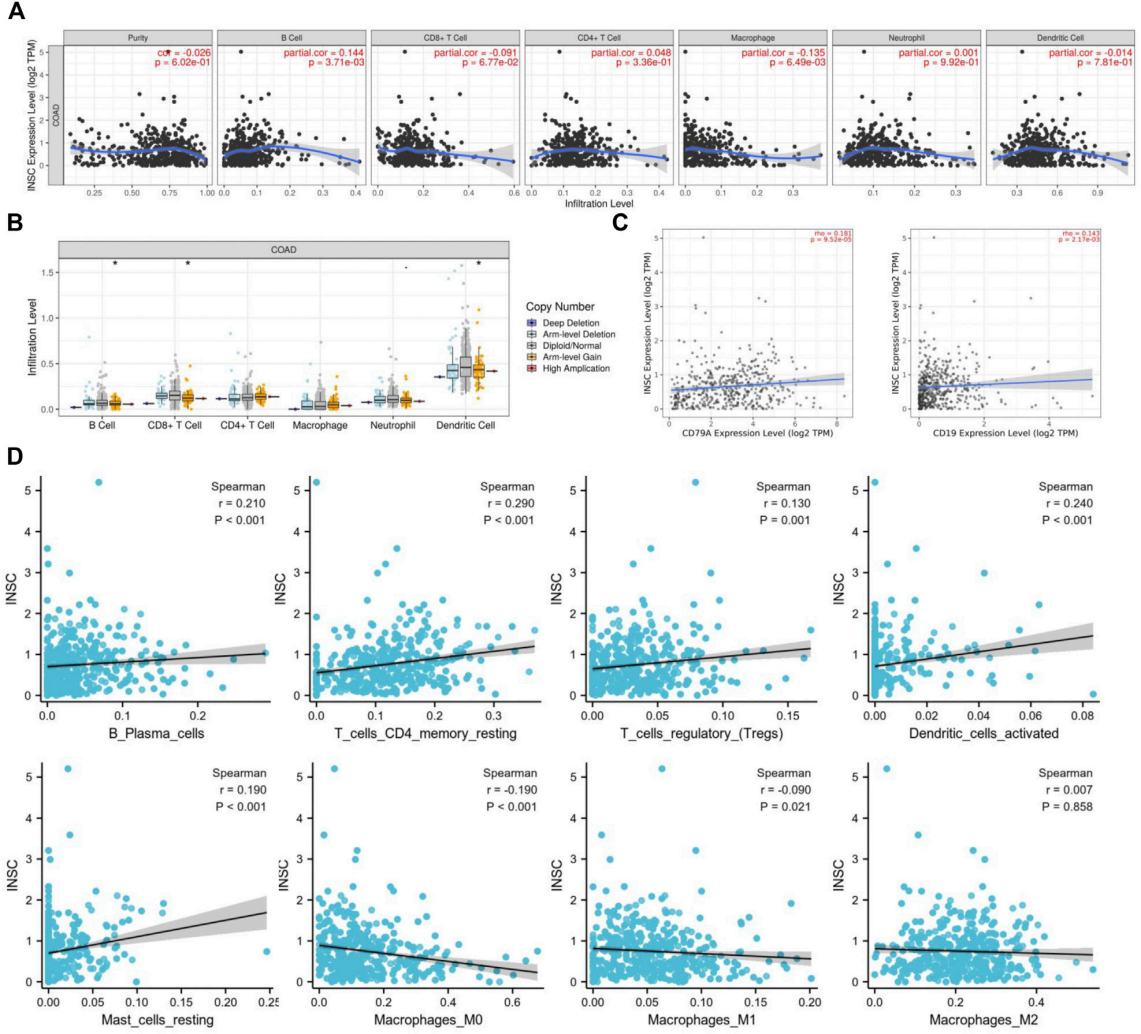
Correlations of INSC expression with TIIC infiltration level in COAD. **(A)** The correlation of INSC expression with tumor purity and TIIC from the TIMER database. **(B)** The effect of INSC CNV on the infiltration levels of immune cells in COAD. **(C)** Relationship between INSC and B cell markers such as CD79A and CD19. **(D)** The effect of INSC on the level of TIIC infiltration in COAD using the CIBERSORT method (*, *p* < 0.05, **, *p* < 0.01, ***, *p* < 0.001).

To further explore how the INSC gene affects tumor progression, the TIMER database was employed to analyze whether INSC expression was associated with TIIC in COAD. The results indicated that the expression of INSC was significantly related to the major immune cell infiltrates, such as B cells and macrophages (Figure 7A). INSC copy number variation (CNV) was correlated with the infiltration levels of B cells, CD8^+^ T cells, and dendritic cells (Figure 7B). There was a positive correlation between INSC and B cell markers, CD19, and CD79A (Figure 7C). In addition, the CIBERSORT method was further used to assess the cellular composition of TIIC in COAD. It suggested statistical significance between INSC and B cells, CD4^+^ T cells, macrophages, and dendritic cells (*p* < 0.05) (Figure 7D). These results suggest that INSC played a critical role in regulating TIIC infiltration in COAD.

### 3.7 Correlation of Inscuteable Spindle Orientation Adaptor Protein Expression With Immune Marker Sets

We focused on the association of INSC with marker genes of diverse immune cells, including CD8^+^ T cells, T cells (normal), B cells, monocytes, TAMs, macrophages M1 and M2, neutrophils, NK cells, and DC, in order to further validate the link between INSC and immune infiltrating cells. INSC expression was shown to be substantially linked with the overwhelming majority of B cell and macrophage immunological markers in COAD. As a result, we were able to demonstrate the COAD microenvironment’s unique relationship with immune infiltrating cells.

## 4 Discussion

As described in the background, it is urgent to understand the molecular mechanism of the genesis and progression of COAD and to explore new markers and therapeutic targets in the context of the high mortality rate and the lack of early diagnosis and effective therapeutic targets. This study aims to explore the diagnostic value of the INSC gene in COAD and its influence on tumor immune infiltration.

Studies have confirmed that INSC is mainly involved in cell mitosis. So far, few studies have paid attention to its role in the occurrence and development of colon cancer. In the present study, we investigated the differential expression of INSC in pan-cancer, including COAD. We discovered that INSC was depressed in a variety of tumor tissues compared to normal tissues as well as in COAD. The immunohistochemical results from 76 COAD patients also confirmed this conclusion. Moreover, INSC mRNA levels were markedly related to pathological T and M stages. Next, the ROC and KM survival analyses were employed to illustrate the relationship between INSC and the survival rate of COAD patients. To ensure the accuracy of the conclusion, we used the data obtained from TCGA and GEO databases at the same time. We further assessed the relationship between INSC expression and clinicopathological features that were related to OS in COAD by univariate and multivariate Cox regression analyses. It revealed that low INSC expression (*p* < 0.001) was an important risk factor for the prognosis of COAD. These results illustrate that low INSC expression is related to worse prognosis, and INSC has potential value in predicting prognosis in COAD.

The PPI network was employed in this investigation to find INSC co-regulatory proteins using string tools. INSC-related genes, such as NUMB and NUMBL, were found to be enriched in the Notch signaling pathway. Notch receptors and their ligands are abnormally active in several forms of human malignancies, including COAD, according to recent research (Suliman et al., 2016). Furthermore, in mammalian cells, dysregulation of the Notch system causes disastrous mitosis (Převorovský et al., 2016). The Notch signaling pathway’s abnormal activation may be involved in the formation of cancers, particularly in colon carcinoma (Zhang et al., 2017). In addition, INSC has been shown to alter mitosis in previous investigations (Wang et al., 2006), and the results obtained in our study are the same. But how INSC affects the Notch signaling pathway remains to be further studied.

An immune infiltration within the tumor often affect the prognosis of patients (Hu et al., 2021; Zhu et al., 2022) and treatment response in many malignancies (Buisseret et al., 2018; Zhu et al., 2021b). So, we focused on INSC-related immune infiltration levels in COAD to learn more about the latent mechanisms of INSC. According to our findings, INSC and B cells, CD4^+^ T cells, macrophages, and dendritic cells showed a substantial difference. INSC expression was positively correlated with B cells, CD4^+^ T cells, and dendritic cell infiltration in COAD, but negatively correlated with M0 and M1 macrophages. According to publications, B cells and CD4^+^ T cells have been related to better clinical outcomes in COAD (Yang et al., 2018). B cell differentiation into antibody-secreting cells is facilitated by the interaction of follicular CD4^+^ T cells with B cells (Wongthida et al., 2020). As the same, contact between CD4^+^ T cells and follicular B cells enhances CD4^+^ T cell activation and differentiation into effector and memory cells (Tay et al., 2019). To sum up, low INSC expression indicates a low amount of B cells and CD4 + T cell infiltration, as well as a poor prognosis.

According to further study, monocytes and macrophages M0 and M1 were also connected with colon cancer survival risk in addition to B-cell naive and B-cell memory (Wu et al., 2020). Several studies demonstrated that macrophages enhance colon cancer cell proliferation, which is consistent with the fact that more macrophages are related to a worse prognosis for colon cancer (Wu et al., 2020). As we know, M1 macrophages and M2 macrophages are two types of macrophages (Han et al., 2020). M1-polarized TAM exerts pro-inflammatory and anticancer effects, whereas M2-polarized macrophages boost carcinogenesis and tumor growth through mediating tumor-promoting immune-suppressive actions in colon cancer (Mühlberg et al., 2016). M2 macrophages predominated in tumor-infiltrating macrophages but M1 macrophages predominated in the non-cancerous inflammatory zone around cancer cell infiltrates (Ino et al., 2013). As a consequence, in this study, we can observe the paradoxical but practical results in Figure 7 and Supplementary Table S1, where INSC as a favorable prognostic marker of COAD has a negative connection with M1 macrophages.

However, there were some limitations to this work, such as the small number of normal samples from the TCGA database and the lack of animal models to test the results. These results will very certainly need to be confirmed in future research. Therefore, cell and animal models will be conducted to further confirm our conclusions through experiments.

## 5 Conclusion

Our findings revealed the correlation of INSC expression levels with the prognosis and clinicopathological features of COAD and the association between INSC expression levels and TIIC infiltration. Thus, our findings imply that INSC expression may have predictive relevance in COAD patients and that it might be a novel target for COAD immunotherapy.

## Data Availability

Publicly available datasets were analyzed in this study. This data can be found here: https://www.cancer.gov/about-nci/organization/ccg/ research/structural-genomics/tcga and the datasets of GEO can be found at: https://www.ncbi.nlm.nih.gov/geo/. The datasets in the current study are also available from the corresponding authors upon reasonable request in compliance with ethical standards.
