# Supplementary material for: INSC Is Down-Regulated in Colon Cancer and Correlated to Immune Infiltration
Source: Front Genet. 2022 May 19;13:821826. doi: 10.3389/fgene.2022.821826 (PMC9161087; doi:10.3389/fgene.2022.821826)
Supplement: Supplementary file 1 [file Table1.PDF]

Characteristic	Low expression of INSC	High expression of INSC	*P*
n	239	239	
Age, n (%)			0.641
<=65	94 (19.7%)	100 (20.9%)	
>65	145 (30.3%)	139 (29.1%)	
Gender, n (%)			0.410
Female	108 (22.6%)	118 (24.7%)	
Male	131 (27.4%)	121 (25.3%)	
T stage, n (%)			0.007
T1	6 (1.3%)	5 (1%)	
T2	29 (6.1%)	54 (11.3%)	
T3	165 (34.6%)	158 (33.1%)	
T4	38 (8%)	22 (4.6%)	
N stage, n (%)			0.235
N0	133 (27.8%)	151 (31.6%)	
N1	60 (12.6%)	48 (10%)	
N2	46 (9.6%)	40 (8.4%)	
M stage, n (%)			0.087
M0	163 (39.3%)	186 (44.8%)	
M1	39 (9.4%)	27 (6.5%)	
Pathologic stage, n (%)			0.030
Stage I	29 (6.2%)	52 (11.1%)	
Stage II	97 (20.8%)	90 (19.3%)	
Stage III	67 (14.3%)	66 (14.1%)	
Stage IV	39 (8.4%)	27 (5.8%)	
CEA level, n (%)			0.234
<=5 ng/mL	89 (29.4%)	107 (35.3%)	
>5 ng/mL	57 (18.8%)	50 (16.5%)	
