# Supplementary material for: INSC Is Down-Regulated in Colon Cancer and Correlated to Immune Infiltration
Source: Front Genet. 2022 May 19;13:821826. doi: 10.3389/fgene.2022.821826 (PMC9161087; doi:10.3389/fgene.2022.821826)
Supplement: Supplementary file 2 [file Table2.PDF]

Description	Gene markers	cor	p.value	pstar
CD8+ T cell	CD8A	-0.073668035	0.116600599	
	CD8B	-0.124686515	0.007750804	**
T cell (general)	CD3D	0.086701107	0.064632524	
	CD3E	0.051220144	0.275592715	
B cell	CD19	0.120948133	0.009814385	**
	CD79A	0.182559698	8.99E-05	**
Monocyte	CD86	-0.128421078	0.006085343	**
	CD115 (CSF1R)	0.015334449	0.744262134	
TAM	CCL2	-0.078822918	0.093085989	
	CD68	-0.157051311	0.000774676	**
M1 Macrophage	INOS (NOS2)	0.368322342	4.58E-16	**
	IRF5	-0.101780641	0.029952103	*
	COX2 (PTGS2)	0.175406254	0.000169611	**
M2 Macrophage	CD163	-0.147404864	0.001616884	**
	VSIG4	-0.144195929	0.002045996	**
	MS4A4A	-0.148487206	0.001491908	**
Neutrophils	CD66b (CEACAM8)	0.237419228	2.99E-07	**
	CD11b (ITGAM)	-0.163995294	0.000444205	**
	CCR7	0.181200783	0.000101602	**
Natural killer cell	KIR2DL1	-0.129727417	0.005583499	**
	KIR2DL3	-0.143904282	0.002089756	**
	KIR2DL4	-0.025287148	0.590580078	
	KIR3DL1	-0.153878101	0.00099147	**
	KIR3DL2	-0.113731379	0.0152167	*
	KIR3DL3	-0.02156437	0.646398055	
	KIR2DS4	-0.108264819	0.020898783	*
Dendritic cell	HLA-DPB1	-0.060989247	0.194089595	
	HLA-DQB1	-0.051860683	0.269625146	
	HLA-DRA	-0.051627296	0.271789172	
	HLA-DPA1	-0.036347881	0.439256288	
	BDCA-1 (CD1C)	0.195352656	2.72E-05	**
	BDCA-4 (NRP1)	-0.129959873	0.005498204	**
	CD11c (ITGAX)	-0.118597578	0.011349325	*
Th1	T-bet (TBX21)	-0.043690174	0.352462325	
	STAT4	0.05229153	0.265661225	
	STAT1	-0.160795149	0.000575573	**
	IFN-γ (IFNG)	-0.099721977	0.033453779	*
	TNF-α (TNF)	0.041596336	0.376036545	
Th2	GATA3	-0.020670741	0.660113761	
	STAT6	0.13709408	0.00338832	**
	STAT5A	-0.081464311	0.082599344	
	IL13	0.048517838	0.301752585	
Tfh	BCL6	-0.160952226	0.000568363	**
	IL21	-0.108120932	0.021070427	*
Th17	STAT3	0.097674851	0.037276115	*
	IL17A	0.354450766	6.47E-15	**
Treg	FOXP3	0.082558373	0.078545192	
	CCR8	0.007619953	0.871231512	
	STAT5B	-0.070999253	0.13048075	
	TGFβ (TGFB1)	-0.042554403	0.365131807	
T cell exhaustion	PD-1 (PDCD1)	-0.051	0.4	
	CTLA4	-0.032	0.6	
	LAG3	-0.056	0.36	
	TIM-3 (HAVCR2)	-0.12	0.052	
	GZMB	-0.024	0.69	
