# Supplementary figures and images for: INSC Is Down-Regulated in Colon Cancer and Correlated to Immune Infiltration
Source: Front Genet. 2022 May 19;13:821826. doi: 10.3389/fgene.2022.821826 (PMC9161087; doi:10.3389/fgene.2022.821826)

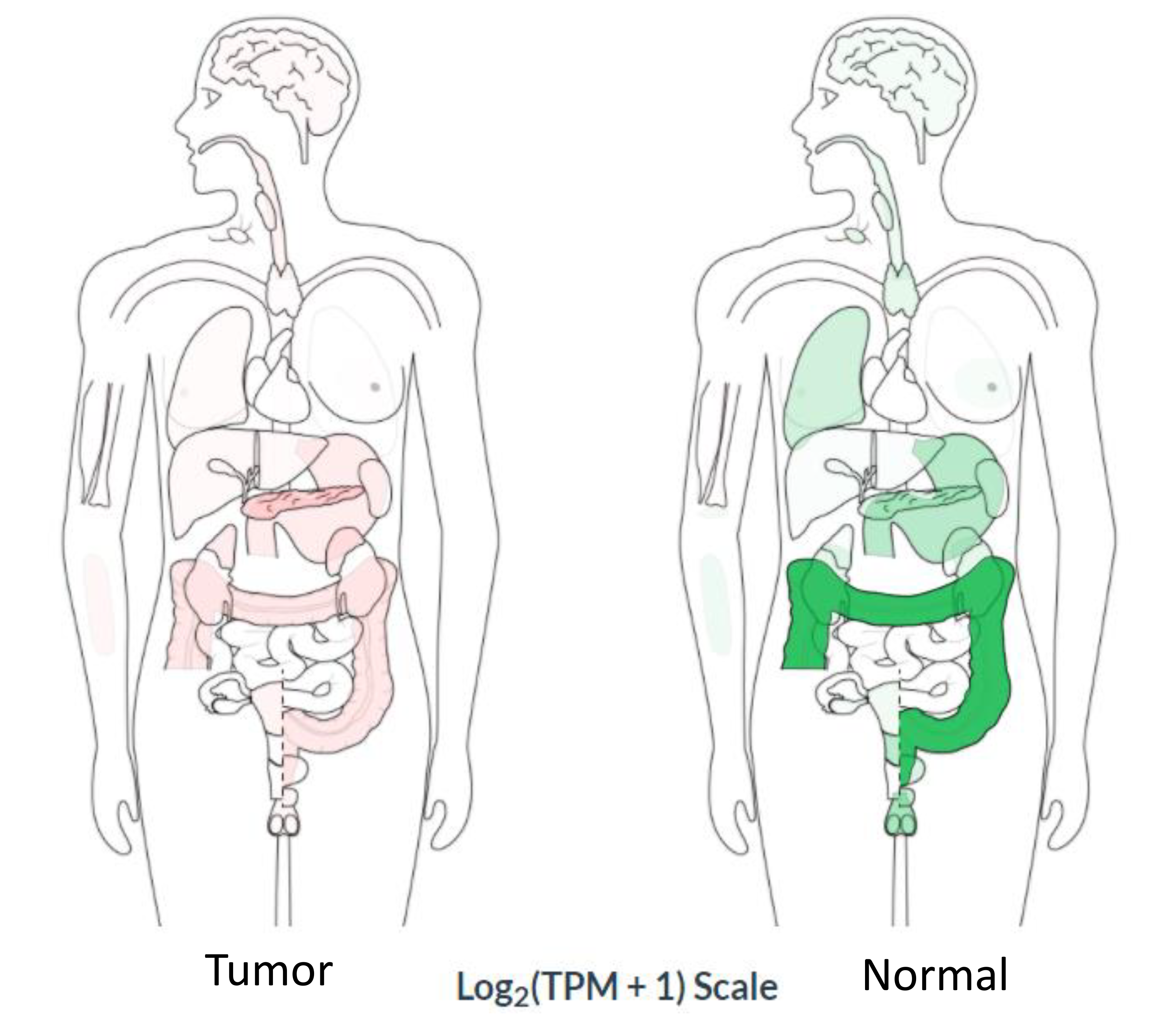

Supplement: Supplementary file 3 [file Image1.TIF]
